# Multivariate logistic regression analysis of the correlation between five biomarkers and ovarian cancer in patients with intermediate-risk: A prospective cross-sectional study

**DOI:** 10.3389/fcell.2022.876071

**Published:** 2022-08-31

**Authors:** Zhen Liu, Jingjing Wu, Xiuli Wang, Xiaoyang Ji

**Affiliations:** ^1^ Department of Nuclear Medicine, Cangzhou Central Hospital, Cangzhou, China; ^2^ Department of Laboratory Medicine, Cangzhou Central Hospital, Cangzhou, China; ^3^ Department of Obstetrics and Gynecology, Ningjin Hospital of Integrated Traditional Chinese and Western Medicine, Xingtai, China

**Keywords:** neutrophil to lymphocyte ratio, platelet to lymphocyte ratio, brain-derived neurotrophic factor, ovarian cancer, logistics regression analysis

## Abstract

**Objective:** To find potential diagnostic biomarkers for ovarian cancer (OC), a prospective analysis of the expression of five biomarkers in patients with intermediate-risk and their correlation with the occurrence of OC was conducted.

**Method:** A prospective observational study was carried out, patients who underwent surgical treatment with benign or malignant ovarian tumors in our hospital from January 2020 to February 2021 were included in this study, and a total of 263 patients were enrolled. Based on the postoperative pathological results, enrolled patients were divided into ovarian cancer group and benign tumor group (n = 135). The ovarian cancer group was further divided into a mid-stage group (n = 46) and an advanced-stage group (n = 82). The basic information of the three groups of patients was collected, the preoperative imaging data of the patients were collected to assess the lymph node metastasis, the preoperative blood samples were collected to examine cancer antigen 125 (CA125), carbohydrate antigen 19–9 (CA19–9), Neutrophil to lymphocyte ratio (NLR), platelet to lymphocyte ratio (PLR), and the postoperative pathological data were sorted and summarized.

**Result:** The average during of disease in the advanced ovarian cancer group was 0.55 ± 0.18 years higher than the benign tumor group (0.43 ± 0.14 years), *p* < 0.001. In the advanced ovarian cancer group, the ratio of patients with the tumor, node, metastasis (TNM) stage IV (64.63%), with tumor Grade stage II and III (93.90%), and without lymph node metastasis (64.63%) was respectively more than that in the mid-stage group (accordingly 0.00, 36.96, 23.91%) (*p* < 0.001); The ratio of patients with TNM grade III in the mid-stage group (73.91%) was more than that in the advanced group (35.37%) (*p* < 0.001). The levels of the five biomarkers: CA19-9, CA125, NLR, PLR, and BDNF were different among the three groups (*p* < 0.001).

**Conclusion:** CA19-9, CA125, NLR, PLR, BDNF are five biomarkers related to the occurrence of ovarian cancer and are risk factors for it. These five biomarkers and their Combined-Value may be suitable to apply in the diagnosis and the identification of ovarian cancer in patients with intermediate-risk.

## 1. Introduction

Ovarian cancer is a common clinical malignant tumor of the female reproductive system. Its incidence is second only to cervical cancer, and it ranks third in the incidence of female malignant tumors. Furthermore, its mortality rate ranks first (Králíčková et al., 2020). Ovarian cancer lacks typical symptoms in the early stage, and most patients seek medical attention for pain, abdominal masses, and ascites. When they are diagnosed, they are in the advanced stage of cancer and have lost the best time for treatment (Jiang et al., 2020). When ovarian cancer progresses to the advanced stage, cancer cells have spread and metastasized, and the 5-years survival rate of patients is about 30%; in contrast, if ovarian cancer patients receive timely treatment in the early stage, the 5-years survival rate of patients would be 70–90% (Lisio et al., 2019). Therefore, exploring the biomarkers related to ovarian cancer is of positive significance for screening ovarian cancer at an early stage. For intermediate-risk patients who have no first-degree relatives diagnosed with ovarian cancer, it is particularly important to clarify which biomarkers are valuable used for ovarian cancer screening and to guide patients to promptly refer to gynecological oncologists based on abnormal indicators and to receive further evaluation (Daly et al., 2021).

Cancer antigen 125 (CA125), one of the tumor markers, is an effective index commonly used in clinical practice. It is used for the diagnosis of ovarian cancer and other malignant tumors, for the evaluation of treatment effects, and for the identification of tumor recurrence (Zhang et al., 2021). Carbohydrate antigen 19-9 (CA19-9) is often used in patients with malignant digestive system tumors, and there was also evidence that it was highly expressed in gynecological diseases (Shinmura et al., 2020). Platelet to lymphocyte ratio (PLR) and neutrophil to lymphocyte ratio (NLR) are newly discovered systemic inflammatory markers in recent years, which can be used to evaluate the prognosis of colorectal cancer, lung cancer, liver cancer and other malignant tumors. It can also effectively identify benign and malignant ovarian tumors and can be used to predict the prognosis of patients (Turkmen et al., 2013). Brain-Derived Neurotrophic Factor (BDNF) is a neurotrophic factor, which is widely distributed in the nervous system and belongs to the family of nerve growth factor proteins. The binding of this protein to its cognate receptor can regulate biological processes, such as stress response and mood disorders, and the latest research suggests that it may be involved in the occurrence and development of ovarian cancer (Okugawa et al., 2013). In the former studies, there are few clinical studies on the application of biomarkers BNDF, NLR, and PLR to the diagnosis of ovarian cancer. Based on this, this study will explore the correlation between the five biochemical markers of CA19-9, CA125, NLR, PLR, BDNF, and other factors and ovarian cancer, and explore the possibility of diagnostic value for ovarian cancer.

## 2. Methods

### 2.1 Population

Patients who underwent surgical treatment of ovarian tumors in our hospital from January 2020 to February 2021, and who met the inclusion and exclusion criteria were enrolled in this prospective observational study. The final postoperative pathological results were used as the final grouping criteria. The in-exclusion criteria are as follows:

The inclusion criteria: (Králíčková et al., 2020) Receive both abdominal and pelvic imaging examinations, and the imaging data, CT and (or) MR, are complete (Jiang et al., 2020); Surgery is required to treat ovarian tumors (Lisio et al., 2019); Ovarian cancer-related gene testing has not been performed (Daly et al., 2021); No anti-tumor treatment before surgery (Zhang et al., 2021); Agree to participate in the study and sign informed consent.

The exclusion criteria: (Králíčková et al., 2020) With a family history of ovarian cancer, or with first- and second-degree relatives diagnosed with ovarian cancer (Jiang et al., 2020); With a history of antibiotic use 2 weeks before surgery (Lisio et al., 2019); Lynch syndrome (Daly et al., 2021); With a history of smoking and blood transfusion (Zhang et al., 2021); Combined infection preoperatively (Shinmura et al., 2020); Data need to be collected are missing (Turkmen et al., 2013); Combined with other gynecological tumors (Okugawa et al., 2013); Combined with digestive system tumors (Javadi et al., 2016); No history of pregnancy or childbirth.

**FIGURE 1:**
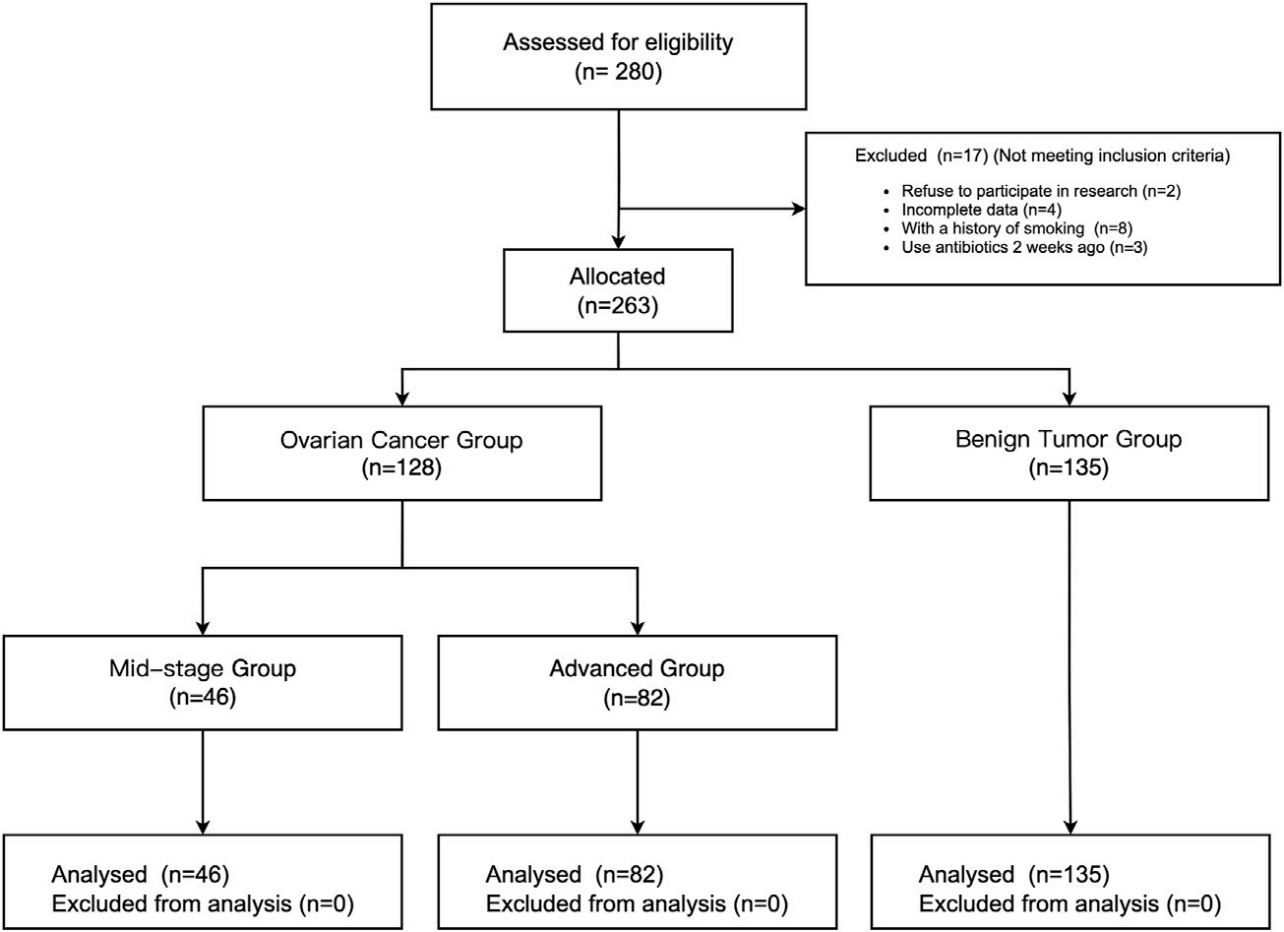
The flow chart of patient enrollment.

From January 2020 to February 2021, a total of 280 patients underwent surgical treatment of ovarian tumors in our hospital, and 17 patients were excluded according to the inclusion and exclusion criteria (2 patients refused to participate, 4 patients with incomplete data, 8 patients with a smoking history, and 3 patients with an antibiotic administration history). The rest of 263 patients were allocated to different groups based on the stage of ovarian tumor (Javadi et al., 2016). 128 patients were included in the ovarian cancer (OC) group, of which 46 were in the mid-stage group and 82 were in the advanced group. 135 patients were included in the benign tumor group. (See Figure 1).

This research complying with the requirements of the “Declaration of Helsinki” was approved by the hospital ethics committee and the informed consent forms were waived.

### 2.2 Study design and outcomes

5–8 ml of fasting venous blood was obtained from the patient in the early morning of the operation day and the blood samples were divided into 2 EP tubes. After centrifugation, the serum was collected from one tube, and the level of CA19-9 and CA125 was detected by enzyme-linked immunosorbent assay (ELISA) using a kit (R&D Systems, TECHNE Corporation, United States); the other tube of the venous blood was sent to the laboratory, and the complete blood count (CBC) was carried out through the automatic blood analyzer (XE-5000, SYSMEX Corporation, Japan). The absolute value of neutrophil, lymphocyte and platelet counts were obtained. Then, the NLR and PLR were calculated. Each sample was measured twice, and the average value was taken as the final value.

The resected ovarian tumor specimens were sent to the pathology department, fixed with 10% formalin, and embedded in paraffin, and then the tumor specimens were sectioned to obtain tissue sections with a thickness of 4 μm. Subsequently, the tissue sections were stained by immunohistochemistry. After immunohistochemical staining, the sections were placed under a 400-fold light microscope for observation, and a high-power field of view with epithelial cells or glandular epithelial cells was randomly selected for image collection and evaluation. The histochemistry score (H-score) method [H-score = ∑Pi(i+1)] (Fardet et al., 2014)was used to evaluate the expression of BDNF in different tissue sections (Lai et al., 2010), where i represents the intensity of cell staining, 0 means no staining, and 1 means yellow, light brown, 2 means brown, 3 means dark brown; Pi represents the proportion of cells of each staining intensity, and the fluctuation range is 0–100%. The final H-score of each slice was the average of 5 high-powered field scores, and an H-score ≥ 2 was considered positive. Each specimen was scored by 2 independent researchers, and the average value of the H-score score was used as the final level of BDNF.

The final pathological result and other clinical data of the patients included in the study were collected after surgery, such as final pathological diagnosis, tumor Grade stage and tumor, node, metastasis (TNM) stage (Gershenwald et al., 2017), tumor involved area, and combined lymph node metastasis. The time between the discovery of abnormal ovarian masses or CA125 abnormalities and the receipt of surgical treatment was used as the duration of disease, and the duration of disease, pregnancy history, and menstrual cycle were collected.

The five biomarkers of CA19-9, CA125, NLR, PLR and BDNF were the primary outcomes, and other data were the secondary outcome. In this study, the value of the result of multiply CA199, CA125, NLR, PLR, and BDNF was taken as a Combined-Value (CV), and independently considered as an index by authors.

### 2.3 Statistics

SPSS 21.0 software (SPSS, Chicago, Illinois, United States) was used for data analysis. CA19-9, CA125, NLR, PLR, and BDNF, these five biomarkers and other continuous measurement data were following the normal distribution after inspection, and the data were expressed as mean ± standard deviation (SD). Analysis of Variance (ANOVA) analysis was performed, and the LSD method was used to perform the inter-group pairwise comparison. The rest of the count data were expressed in the form of a ratio, and the R*C chi-square test was performed for statistical analysis.

The five biochemical markers of CA19-9, CA125, NLR, PLR and BDNF and the CV were analyzed by Spearman correlation with the occurrence of ovarian cancer, the R value and *p* value were obtained. Finally, the statistically significant parameters in the analysis of ANOVA analysis, chi-square test and Spearman correlation analysis were included in the multivariate Logistic regression analysis, the stepwise regression method was used to perform the multivariate Logistic regression analysis to obtain the regression coefficient, odds ratio (OR) value and *p* value (stepwise regression method, entry: *p* = 0.05; removal *p* = 0.10). The linear model was used to diagnose the collinearity of the variables. Logistic regression analysis results are expressed as OR value, 95% confidence interval (95% CI), *p* value. All statistical results are regarded as statistically significant with *p* < 0.05.

## 3. Results

### 3.1 General information comparison

A total of 263 patients were included in this study. The benign tumor group (n = 135) and the ovarian cancer group were further divided into the mid-stage group (n = 46) and the advanced group (n = 82) based on the pathological results.

There was no significant difference in age, tumor involved area, pregnancy history, and menstrual cycle among the groups (*p* > 0.05); the average during of disease in the advanced ovarian cancer group was 0.55 ± 0.18 years, which was higher than the benign tumor group of 0.43 ± 0.14 years, the difference was statistically significant, *p* < 0.001. The duration of disease of the mid-stage ovarian cancer group was 0.47 ± 0.15 years, compared with the benign tumor group, the difference was not statistically significant (*p* > 0.05). The number of patients in TNM-IV stage and Grade stage II-III in the advanced group, and the number of patients with lymph node metastases were more than those of the mid-stage group, and the difference was statistically significant (*p* < 0.05). There are also differences in the pathological results between ovarian cancer and benign tumor group, and the differences are statistically significant (*p* < 0.05). (See Table 1).

**TABLE 1:** | General information comparison.

**Items**	**Ovarian Cancer Group N = 128**		**Benign Tumor Group** **N = 135**	**Statistics**	***p* -Value**
	**Mid-stage group N = 46**	**Advanced group N = 82**			
Age (years)	50.98 ± 9.59	50.78 ± 9.87	50.87 ± 9.93	0.006	0.994
Duration of disease (years)	0.47 ± 0.15	0.55 ± 0.18	0.43 ± 0.14	15.270	<0.001*^a^
**TNM stage**					
TNM-I	0	0	–	–	–
TNM-II	12 (26.09%)	0 (0.00%)	–	23.604	<0.001*
TNM-III	34 (73.91%)	29 (35.37%)	–	17.519	<0.001*
TNM-IV	0 (0.00%)	53 (64.63%)	–	50.742	<0.001*
**Final pathology result**				263.313	<0.001*
Serous cystadenocarcinoma	24 (52.17%)	42 (51.22%)	0 (0.00%)		
Mucinous cystadenocarcinoma	11 (23.91%)	18 (21.95%)	0 (0.00%)		
Endometrioid carcinoma	7 (15.22%)	14 (17.07%)	0 (0.00%)		
Poorly differentiated adenocarcinoma	4 (8.70%)	8 (9.76%)	0 (0.00%)		
Serous cystadenoma	0 (0.00%)	0 (0.00%)	52 (38.52%)		
Mature teratoma	0 (0.00%)	0 (0.00%)	42 (31.11%)		
Mucinous cystadenoma	0 (0.00%)	0 (0.00%)	33 (24.44%)		
Ovarian Fibroma	0 (0.00%)	0 (0.00%)	8 (5.93%)		
**Tumor involved area**				2.648	0.266
unilateral	9 (19.57%)	14 (17.07%)	15 (11.11%)		
bilateral	37 (80.43%)	68 (82.93%)	120 (88.89%)		
**Grade classification**				48.989	<0.001*
Grade I	29 (63.04%)	5 (6.10%)	–		
Grade II-III	17 (36.96%)	77 (93.90%)	–		
**Lymph node metastasis**				19.546	<0.001*
With	35 (76.09%)	29 (35.37%)			
Without	11 (23.91%)	53 (64.63%)			
Pregnancy history (times)	2.59 ± 0.65	2.49 ± 0.59	2.51 ± 0.61	0.417	0.659
Menstrual cycle (days)	28.21 ± 5.76	28.32 ± 5.69	28.19 ± 5.82	0.013	0.987

*p < 0.05, the difference is statistically significant.

a the difference between Advanced group and Benign tumor Group has statistically significant. TNM: tumor, node, metastasis; The TNM stage of Malignant Tumors.

### 3.2 Comparison of the levels of CA19-9, CA125, NLR, PLR, and BDNF in each group

The levels of CA19-9, CA125, NLR, PLR, and BDNF in the advanced group were higher than those in the mid-stage and benign tumor group. The levels of these 5 biochemical markers in the mid-stage group were also higher than those in the benign tumor group. The differences were statistically significant (*p* < 0.05). (See Table 2).

**TABLE 2:** | Comparison of the levels of CA19-9, CA125, NLR, PLR, and BDNF in each group.

**Items**	**Ovarian Cancer N = 128**		**Benign Tumor Group** **N = 135**	**Statistics**	***p*-Value**
	**Mid-stage group N = 46**	**Advanced group N = 82**			
CA19-9 (U/mL)	67.98 ± 20.98	89.87 ± 21.19	24.39 ± 2.38	530.190	<0.001*^abc^
CA125 (U/mL)	675.98 ± 76.92	934.92 ± 73.09	31.09 ± 11.21	8202.824	<0.001*^abc^
NLR (%)	3.27 ± 0.38	4.12 ± 0.32	2.03 ± 0.29	1149.902	<0.001*^abc^
PLR (%)	136.63 ± 18.98	198.93 ± 19.23	87.94 ± 17.63	932.891	<0.001*^abc^
BDNF (H-Score)	2.26 ± 0.23	2.93 ± 0.21	1.31 ± 0.19	1665.211	<0.001*^abc^

*p < 0.05, the difference is statistically significant. Data were expressed as Mean ± Standard Deviation

a the difference between Mid-stage group and Benign tumor Group has statistically significant.

b the difference between Advanced group and Benign tumor Group has statistically significant.

c the difference between Mid-stage group and Advanced group has statistically significant.

CA125, cancer antigen 125; CA19–9, carbohydrate antigen 19–9; NLR, Neutrophil to Lymphocyte Ratio; PLR, Platelet to Lymphocyte Ratio; BDNF, Brain-Derived Neurotrophic Factor; H-Score, histochemistry score.

### 3.3 The correlation between primary outcomes and partial secondary outcomes and the ovarian cancer

According to the result of Spearman correlation analysis, lymph node metastasis (R = 0.479, *p* = 0.012), CA19-9 (R = 0.323, *p* = 0.023), CA125 (R = 0.287, *p* = 0.031) NLR (R = 0.304, *p* = 0.025), PLR (R = 0.563, *p* = 0.037) BDNF (R = 0.491, *p* = 0.018) and the CV (R = 0.671, *p* = 0.004) were positively correlated with the occurrence of ovarian cancer.

### 3.4 Multivariate logistic regression analysis of risk factors related to ovarian cancer

CA19-9, CA125, NLR, PLR, BDNF, lymph node metastasis and the CV were all risk factors for ovarian cancer. The OR value was greater than 1, and the *p* value was less than 0.05 (See Table 3).

**TABLE 3:** | Multivariate Logistic Regression Analysis of Risk Factors Related to Ovarian Cancer.

**Items**	**Regression coefficients**	**The Standard deviation**	**Wald Value**	**Odds Ratio**	**95% CI**	***p*-Value**
Duration of disease	0.318	0.602	0.351	0.219	(0.067, 1.688)	0.089
TNM-III	0.308	0.271	6.201	0.802	(0.609, 2.098)	0.107
TNM-IV	0.578	0.873	0.473	0.727	(0.723, 0.529)	0.085
With lymph node metastasis	0.381	0.319	3.091	1.379	(1.009, 1.893)	0.021*
CA19-9	0.828	0.297	4.903	1.652	(1.012, 2.868)	<0.001*
CA12-5	0.265	0.312	2.675	1.522	(0.565, 2.076)	0.027*
NLR	0.332	0.113	8.721	1.372	(1.189, 1.572)	<0.001*
PLR	0.752	0.297	6.287	1.698	(1.287, 2.387)	0.021*
BDNF	0.641	0.961	0.469	2.796	(2.001, 3.654)	0.003*
Combined-Value	0.109	0.312	9.551	3.428	(2.008, 4.398)	<0.001*

*p < 0.05, the difference is statistically significant.

95% CI, 95% Confidence Interval; TNM, tumor, node, metastasis; The TNM stage of Malignant Tumors; CA125, cancer antigen 125; CA19–9, carbohydrate antigen 19–9; NLR, Neutrophil to Lymphocyte Ratio; PLR, Platelet to Lymphocyte Ratio; BDNF, Brain-Derived Neurotrophic Factor; Combined-Value, It is defined by author that Combined-Value is equal to the result of multiplying five factors (CA19-9, CA12-5, NLR, PLR, BDNF).

## 4. Discussion

Research shows that ovarian cancer is prevalent worldwide, with about 225,000 new cases and 140,000 deaths every year. The prognosis of patients in the advanced stage is poor, and patients are prone to recurrence after treatment. The 5-years survival rate of ovarian cancer patients is significantly lower than that of other malignant tumors (Liu et al., 2020; Murakami et al., 2020). Therefore, early diagnosis, early detection, and timely and effective treatment are extremely important for ovarian cancer patients. At present, the clinical treatment of ovarian cancer is still based on surgery, supplemented by radiotherapy, chemotherapy, and immunotherapy (Roett and Evans, 2009). However, most patients have progressed to the middle and advanced stages when they see a doctor. Even after standard treatments such as surgery, radiotherapy, and chemotherapy, the probability of tumor recurrence or metastasis is still high (Stewart et al., 2019). Previous evidence points out that if early ovarian cancer can be detected in time and given effective treatment, the survival rate can be as high as 90% (Nebgen et al., 2019). Therefore, early diagnosis is very important to improve the survival cycle of ovarian cancer patients. At present, there are no precise biomarkers for the diagnosis of ovarian cancer. The current mainstream screening method is to screen the results of CA12-5 combined with the results of transvaginal ultrasound imaging. This screening method should be widely used in high-risk patients (Zhang et al., 2021). This study focused on the middle-risk population, taking patients with benign and malignant ovarian tumors undergoing surgical treatment as the research population to explore the correlation between five biochemical markers and ovarian cancer. This study also created a combined value and used the product of five as an indicator to explore for the next step in diagnostic screening for ovarian cancer, as well as for ovarian cancer recurrence monitoring and referral to gynecological oncologists for further evaluation and exploration the way. The results of this study show that lymph node metastasis, CA19-9, CA125, NLR, PLR, BDNF and the CV are all risk factors for ovarian cancer.

CA125 was a macromolecular glycoprotein developed by Bast et al., in 1981 (Bocheva et al., 2015). It is expressed in the epithelial tissue of the body cavity. It can be used for the auxiliary diagnosis of certain malignant tumors such as ovarian cancer and endometrial cancer and monitor the changes in the condition after treatment (Jeerakornpassawat and Suprasert, 2020). Studies have shown that CA 125 can predict ovarian cancer, but its effectiveness was affected by its limited specificity and very low positive predictive value (PPV). A prospective study found that an increase in CA 125 of more than 30 units/ml was a predictor of subsequent ovarian cancer risk (Jacobs et al., 1996). CA 125 varies with race and smoking status, and it was also elevated in approximately 1% of healthy women and was affected by the menstrual cycle, and increases with age (Partridge et al., 2009). Most importantly, in the PLCO cancer screening test, 74% of ovarian cancers detected by CA 125 are at an advanced stage (stage IIIC or IV). The CA125 screening test did not reduce the mortality rate caused by ovarian cancer (Partridge et al., 2009). Therefore, although CA125 is still the main indicator of ovarian cancer screening, the application of CA125 alone is still not satisfactory. In this study, the relationship between CA125 and ovarian cancer has reached a consistent conclusion with the previous studies. It once again proved that CA125 was related to ovarian cancer, and the increase in CA125 indicates the occurrence of ovarian cancer.

CA19-9 is an oligosaccharide antigen, the level in tumor tissues far exceeds that in normal tissues. It has significant clinical significance for the occurrence of malignant tumors and cell differentiation (Shinmura et al., 2020). CA19-9 is distributed in many parts of the body. It is commonly found in the pancreas and bile duct epithelium of normal adults. It is mostly used for the diagnosis and monitoring of pancreatic cancer (Ge et al., 2017). It has high sensitivity and specificity. CA 19-9 is mainly used to monitor the response of the disease to treatment or to detect the recorded recurrence of gastric cancer (Shen et al., 2018). It is rarely used in the treatment of ovarian cancer. But it may elevate in ovarian cancer. Studies have shown that increased levels of CA19-9 are related to the female gender and the presence of lymph node metastasis (Canney et al., 1985). The level of CA19-9 in the ovarian cancer group was significantly higher than that in the benign tumor group and it should be paid attention to.

Inflammation increases the risk and progression of cancer and is known to play an important role in tumorigenesis, including initiation, promotion, malignant transformation, invasion, and metastasis (Djordjevic et al., 2018). Many inflammatory markers are related to cancer progression and prognosis (Pinto et al., 2018). CBC is a basic preoperative laboratory assessment, and NLR and PLR can be derived easily and quickly using CBC results. NLR was useful as a strong prognostic factor for a variety of cancers (Templeton et al., 2014). Studies have shown that the normal value of NLR for healthy adults and non-elderly groups was between 0.78 and 3.53. However, the study did not include patients with gynecological diseases (Forget et al., 2017). Studies have also shown that NLR greater than 2.6 can better predict the occurrence of ovarian cancer (Raungkaewmanee et al., 2012). In this study, the level of NLR in the middle and advanced ovarian cancer group was higher than that in the benign tumor group, and both were greater than 2.6, again proving the effectiveness of this parameter of NLR in screening for ovarian cancer. Platelets are part of the inflammatory response, including factors related to tumor growth, invasion, and angiogenesis (Chon et al., 2021). Although few published studies have shown that PLR was an independent prognostic marker for ovarian cancer, one study used ROC analysis and obtained the best PLR cutoff value at 200 (Chon et al., 2021). The PLR value of the advanced ovarian cancer group in this study was also closer to the cut-off value of 200 in previous studies. This study suggested that NLR and PLR were related to the occurrence of ovarian cancer, and it was worth paying attention to the increase in the ratio of these two when ovarian cancer was suspected.

BDNF is a member of the growth factor family of neurotrophic factors (Kang et al., 2020a). BDNF exerted its effects by binding to the tropomyosin receptor kinase B (TrkB) receptor that regulates neuron survival and differentiation (Kang et al., 2020a). Although originally thought to be expressed in neuronal tissues, BDNF and TrkB had also been found in various normal non-neuronal tissues in adults (Kang et al., 2020b). In addition to its distribution in normal tissues, BDNF and/or TrkB were also found in tumors. BDNF had been found in neuroblastoma, and TrkB had been shown to mediate chemotherapy resistance (Choi et al., 2016; Xu et al., 2019; Kang et al., 2020b). TrkB and/or BDNF had also been found in other solid malignancies, such as pancreatic ductal adenocarcinoma (Sakamoto et al., 2001), prostate cancer (Li et al., 2020), and lung cancer (Wessels et al., 2015). Studies had shown that BDNF in follicular fluid stimulates fallopian tube epithelial cells that TrkB to promote their survival, migration, and attachment, leading to ovarian cancer (Kang et al., 2020a). A study had also shown that the co-expression of BDNF and TrkB was associated with the poor prognosis of small cell lung cancer (SCLC) patients. BDNF promoted the migration of SCLC cells overexpressing TrkB (Wessels et al., 2015). BDNF was mainly expressed in the cytoplasm of epithelial cells or glandular epithelial cells (Choi et al., 2016). In this study, the specimen sections were stained by immunohistochemistry and then observed under an optical microscope. H-score was used to evaluate the expression of BDNF in different tissue sections. In this study, the average BDNF H-score of the patients in the ovarian cancer group were greater than 2 points, suggesting positive result, which was statistically different from the benign tumor group. It suggested that BDNF may become a biomarker for molecular diagnosis, targeted therapy, and prognostic evaluation of ovarian cancer. However, the detection method of BDNF in this study makes the results of this study on BDNF not directly applicable to clinical screening for ovarian cancer. Studies had shown that BDNF can be detected in serum by ELISA, which was a step further from applying BDNF to ovarian cancer screening (Naegelin et al., 2018).

This study also has some limitations: 1) The screening of ovarian cancer risk factors of the included patients was not detailed, the use of contraceptives and menarche were not evaluated; 2) The ultrasound examination results are greatly affected by the operator, so the ovarian-related ultrasound imaging results of the included patients were not analyzed, and the description of tumor size and other information about the tumor were also lacking; 3) The age of the patients was not distinguished, and young patients were included, which may bias the results; 4) There was no analysis of the postoperative results about the biomarkers.

## Conclusion

In summary, CA19-9, CA125, NLR, PLR, and BDNF were all highly expressed in ovarian cancer patients and were related to the occurrence of ovarian cancer. CA19-9, CA125, NLR, PLR, BDNF and their CV were all risk factors for ovarian cancer. It is possible to consider applying CA19-9, CA125, NLR, PLR, BDNF and their CV to the screening of patients with inter medium-risk ovarian cancer in the future, which may guide the monitoring of ovarian cancer recurrence and guide patients to timely referral to gynecological oncologists for comprehensive evaluate.

## Data Availability

The original contributions presented in the study are included in the article/Supplementary Material, further inquiries can be directed to the corresponding author.

## Abbreviations

BDNF, brain-derived neurotrophic factor; CA125, cancer antigen 125; CA19–9, carbohydrate antigen 19–9; CV, Combined-Value; CBC, complete blood count; ELISA, enzyme-linked immunosorbent assay; H-SCORE, histochemistry score; NLR, Neutrophil to lymphocyte ratio; OC, ovarian cancer; OR, odds ratio; PLR, platelet to lymphocyte ratio; TrkB, tropomyosin receptor kinase B.
